# Distinct antibody clones detect PD-1 checkpoint expression and block PD-L1 interactions on live murine melanoma cells

**DOI:** 10.1038/s41598-022-16776-1

**Published:** 2022-07-21

**Authors:** Christina Martins, Mariana Silva, Erik Rasbach, Praveen Singh, Yuta Itoh, Jason B. Williams, Edith Statham, Anna Meurer, Daniela V. Martinez, Anne Brandenburg, Markus V. Heppt, Steven R. Barthel, Tobias Schatton

**Affiliations:** 1 Department of Dermatology, Harvard Skin Disease Research Center, Brigham and Women’s Hospital, Harvard Medical School, HIM Building, Suite 671, 77 Avenue Louis Pasteur, Boston, MA 02115 USA; 2 Department of Surgery, University Hospital Mannheim, Heidelberg University, 68167 Mannheim, Germany; 3 Department of Dermatology and Allergology, University Hospital Bonn, 53127 Bonn, Germany; 4 Department of Dermatology, University Hospital Erlangen, Friedrich-Alexander-University (FAU) Erlangen-Nuremberg, 91054 Erlangen, Germany; 5 Department of Medicine, Boston Children’s Hospital, Harvard Medical School, Boston, MA 02115 USA

**Keywords:** Melanoma, Immunotherapy

## Abstract

Monoclonal antibodies (abs) targeting the programmed cell death 1 (PD-1) immune checkpoint pathway have revolutionized tumor therapy. Because T-cell-directed PD-1 blockade boosts tumor immunity, anti-PD-1 abs have been developed for examining T-cell-PD-1 functions. More recently, PD-1 expression has also been reported directly on cancer cells of various etiology, including in melanoma. Nevertheless, there is a paucity of studies validating anti-PD-1 ab clone utility in specific assay types for characterizing tumor cell-intrinsic PD-1. Here, we demonstrate reactivity of several anti-murine PD-1 ab clones and recombinant PD-L1 with live B16-F10 melanoma cells and YUMM lines using multiple independent methodologies, positive and negative PD-1-specific controls, including PD-1-overexpressing and PD-1 knockout cells. Flow cytometric analyses with two separate anti-PD-1 ab clones, 29F.1A12 and RMP1-30, revealed PD-1 surface protein expression on live murine melanoma cells, which was corroborated by marked enrichment in PD-1 gene (*Pdcd1*) expression. Immunoblotting, immunoprecipitation, and mass spectrometric sequencing confirmed PD-1 protein expression by B16-F10 cells. Recombinant PD-L1 also recognized melanoma cell-expressed PD-1, the blockade of which by 29F.1A12 fully abrogated PD-1:PD-L1 binding. Together, our data provides multiple lines of evidence establishing PD-1 expression by live murine melanoma cells and validates ab clones and assay systems for tumor cell-directed PD-1 pathway investigations.

## Introduction

The programmed cell death 1 (PD-1) receptor is a premier immune checkpoint target for cancer immunotherapy^1^. Indeed, four separate anti-PD-1 blocking antibody (ab) clones, nivolumab, pembrolizumab, cemiplimab, and dostarlimab, have been approved by the FDA for the treatment of advanced stage cancers of diverse origin, including the first two for melanoma^2^. By inhibiting T-cell-PD-1 interactions with its ligands, PD-L1 and PD-L2, these abs promote antitumor immunity and prolong survival in patients with metastatic disease^3^. Similarly, in preclinical cancer models, several PD-1 blocking ab clones, including 29F.1A12, thwart tumor progression^4–8^. Because T-cell-directed PD-1 inhibition is critical for achieving robust therapeutic benefit, both clinical and experimental studies of the PD-1 pathway have predominantly focused on PD-1 ab effects on T-cell immunobiology^1^. However, PD-1 expression is not restricted to T-cells. In fact, PD-1 has also been identified on multiple additional immune and non-immune cell types within the tumor microenvironment (TME)^9^, and its inhibition on these cells might thus factor into therapeutic outcomes.

For example, TME-infiltrating macrophages and natural killer (NK) cells both express PD-1, the blockade of which by 29F.1A12 contributes to tumor growth suppression and prolonged survival^10,11^. PD-1 has also been identified directly on cancer cells in multiple tumor entities, such as melanoma^5,7,12–19^, non-small cell lung^17,20,21^, colorectal^18,19,21^, brain^22,23^, liver^18,24–26^, pancreatic^17,27^, gastric^28^, esophageal^29–31^, and thyroid cancers^32,33^. In preclinical human and murine melanoma models, including B16-F10, PD-1 functions as a tumor cell-intrinsic receptor that promotes tumorigenesis and metastatic dissemination^5,7^. 29F.1A12 recognizes PD-1 on B16-F10 and other murine tumor lines, suppresses cancer cell-intrinsic PD-1 receptor signaling, and inhibits resultant tumor growth in three-dimensional (3D) culture and tumor-bearing mice^5,7^. Despite multiple independent studies demonstrating PD-1 functional expression by live B16-F10 cells^5,7,12,15,16^, multiple additional tumor, and non-T immune cell types^10,11^, one report^34^ claims that “B16-F10 melanoma cells do not express PD-1” and that “PD-1 expression by non-T-cells is unlikely to be the case”. The authors further allege that 29F.1A12 reacts with dead, but not live, B16-F10 or non-T immune cells^34^. These conclusions are inconsistent with findings by multiple independent groups and are based on suboptimal experimental conditions, including the use of > 10-fold lower PD-1 ab concentrations compared to published reports^5,7^.

Here, we rigorously validate tumor cell-intrinsic PD-1 functional expression by live B16-F10 melanoma cells and six distinct YUMM lines^35,36^ using multiple anti-PD-1 ab clones, recombinant (r)PD-L1, numerous negative and positive control cells of defined PD-1 expression level, and several independent assay systems. Quantitative real-time RT-PCR (qRT-PCR) using two independent primer sets revealed PD-1 gene (*Pdcd1*) expression in wild-type (WT) B16-F10 and YUMM cells, PD-1-overexpressing (OE) B16-F10 melanoma variants, unactivated and CD3/CD28-activated WT positive, but not PD-1 knockout (KO) negative, control B16-F10 or C57BL/6 T-cells. Consistently, immunoblotting using anti-PD-1 ab, AF1021, immunoprecipitation (IP) with clone RMP1-14, and/or mass spectrometric (MS) sequencing of IP products confirmed PD-1 protein expression in WT and PD-1 OE B16-F10 cells, and in WT unactivated and activated T-cells. Flow cytometric analyses using two additional, widely employed anti-PD-1 ab clones, 29F.1A12 and RMP1-30, counterstaining with fixable viability dye (FVD), and isotype-matched ab-controlled gating strategies confirmed PD-1 surface protein expression on live WT B16-F10, YUMM, and PD-1 OE B16-F10 cells, unactivated and activated WT T-cells, but not PD-1 KO B16-F10 or T-cells. Consistently, *Pdcd1* expression was markedly enriched in 29F.1A12 and RMP1-30 FACS-sorted PD-1^+^ versus PD-1^−^ live WT B16-F10 and activated T-cell cohorts. Recombinant PD-L1 also recognized melanoma cell-expressed PD-1 in live B16-F10 cells, as corroborated by significantly enhanced *Pdcd1* expression in rPD-L1-bound versus -non-reactive FACS-sorted cell subsets. Finally, PD-1:PD-L1 binding to both WT and PD-1 OE B16-F10 cells was abrogated by neutralizing^4,8^ 29F.1A12, but not non-blocking^37^ RMP1-30. Together, our data rigorously establishes PD-1 expression by live B16-F10 and YUMM melanoma cells. We validate PD-1 ab clone and rPD-L1 utility in multiple assay systems under defined experimental conditions for detecting tumor cell-intrinsic PD-1 expression and dissecting its function.

## Results

### B16-F10 melanoma cells express PD-1

**Figure 1:**
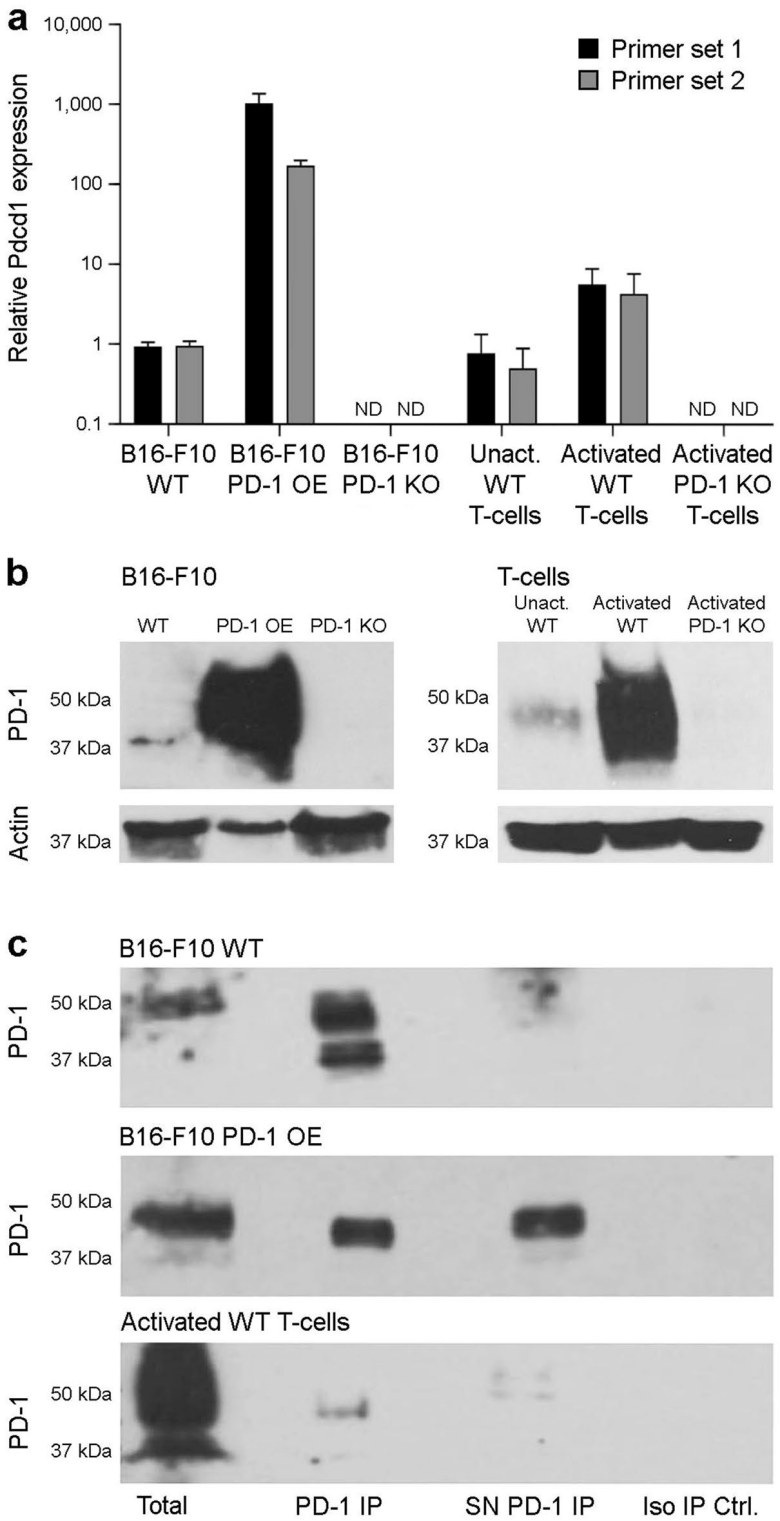
PD-1 is expressed by murine B16-F10 melanoma cells. (**a**) Relative PD-1 gene (*Pdcd1*) expression (mean ± s.d., two distinct primer sets) in wild-type (WT), PD-1-overexpressing (OE), and PD-1 knockout (KO) murine B16-F10 melanoma cells, and in unactivated and activated WT, and PD-1 KO T-cells from C57BL/6 mice, as determined by real-time quantitative RT-PCR. (**b**) Immunoblot of PD-1 protein expression and respective β-actin loading controls in cells as above. (**c**) Immunoprecipitation (IP) of PD-1 (lane 2) from B16-F10 WT (top), B16-F10 PD-1-OE melanoma (center), and activated WT T-cell (bottom) lysates and respective supernatants (SN, lane 3). PD-1 immunoblots of total protein (lane 1) and isotype IP negative controls (lane 4) are also depicted. PD-1 protein identity for immunoprecipitated bands was confirmed by mass spectrometry (MS)-based sequencing. All results are representative of at least *n* = 3 independent experiments. ND, not detected. Please see Supplementary Information for full-length Western blot images.

We first validated our previous findings of *Pdcd1* expression in murine B16-F10 melanoma cells^5^ by real-time qPCR, using two independent *Pdcd1* primer sets and positive and negative control cells of varying PD-1 expression level. As already shown previously^5,15,16^, B16-F10 wild-type (WT) cells expressed marked levels of *Pdcd1* (qPCR cycle threshold ≤ 25 for both primer sets) that did not substantially differ from those in positive control unactivated syngeneic (C57BL/6) T-cells (Fig. 1a). As expected, *Pdcd1* expression was > 100-fold increased in PD-1 OE and > 4-fold in CD3/CD28-activated T-cells compared to WT B16-F10 melanoma cells, but not detected in negative control, PD-1 KO B16-F10 or activated T-cells (Fig. 1a), thus confirming specificity of both primer sets for *Pdcd1*. Immunoblotting corroborated PD-1 protein expression by B16-F10 WT and PD-1 OE, but not PD-1 KO melanoma cells, and by unactivated and activated WT, but not PD-1 KO T-cells at an expected molecular weight of ~ 37–50 kDa (Fig. 1b), consistent with previous studies^5,15^. Anti-PD-1, but not isotype control ab IP also revealed a predominant band at ~ 50 kDa, and an additional band at ~ 37 kDa, in WT and PD-1 OE B16-F10 melanoma cells, and in activated T-cells (Fig. 1c). PD-1 protein identity was verified in IP eluates by MS sequencing. Together, these results rigorously confirm PD-1 transcript and protein expression by B16-F10 melanoma cells.

### The anti-PD-1 29F.1A12 monoclonal antibody recognizes PD-1 surface protein on live B16-F10 melanoma cells

**Figure 2:**
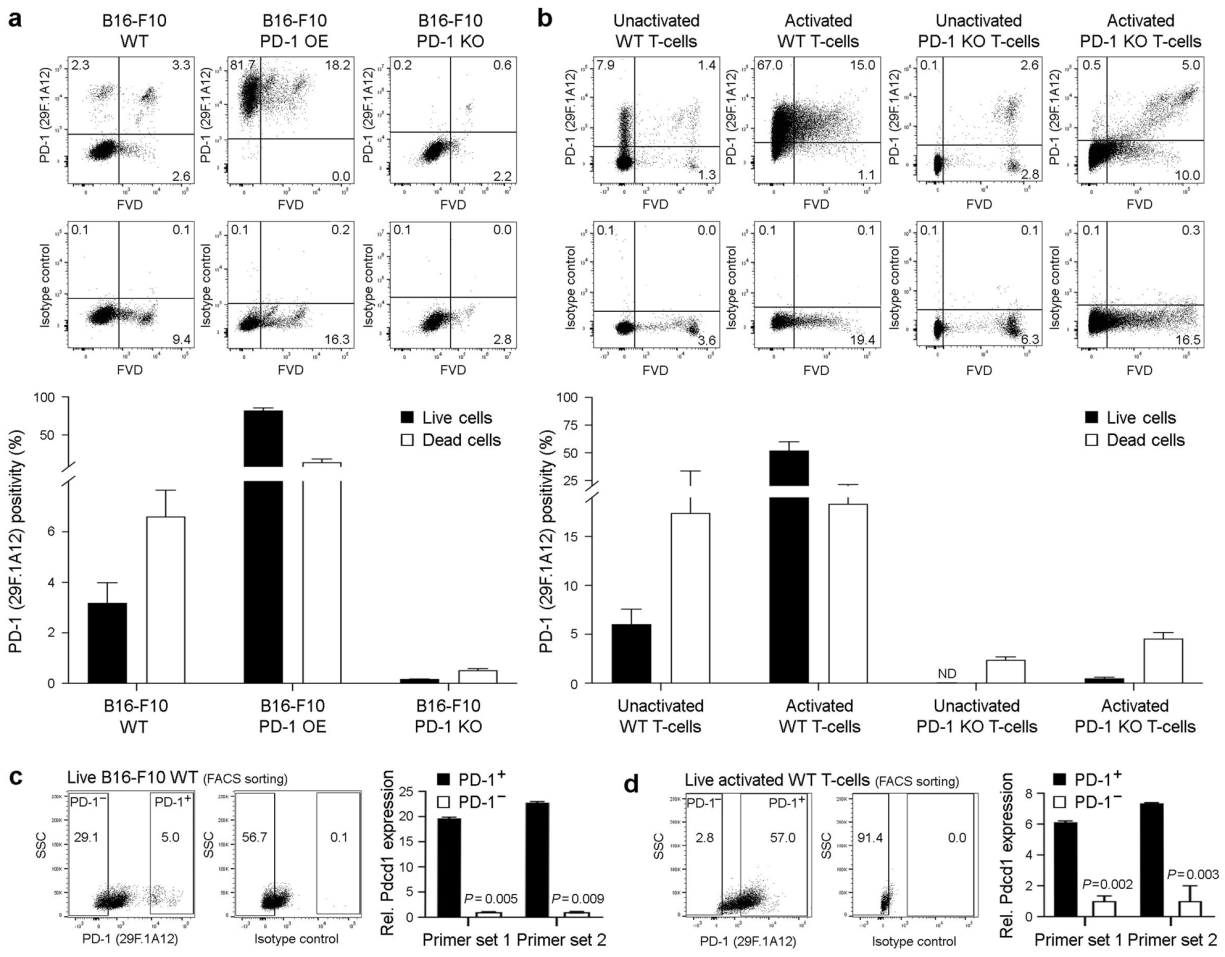
The anti-PD-1 29F.1A12 monoclonal antibody recognizes PD-1 surface protein on live B16-F10 melanoma cells. (**a,b**) Representative FACS plots of surface PD-1 protein expression (clone 29F.1A12) or isotype control staining and % positivity (mean ± s.e.m.) on live versus dead (fixable viability dye, FVD-negative or -positive, respectively) (**a**) B16-F10 wild-type (WT), PD-1-overexpressing (OE), or PD-1 knockout (KO) melanoma cells, or on (**b**) unactivated versus activated WT or PD-1 KO T-cells (C57BL/6), respectively. (**c,d**) Relative Pdcd1 expression (mean ± s.d., two distinct primer sets), as determined by real-time qPCR (right), in FACS-sorted (29F.1A12, left) live (FVD^−^) (**c**) PD-1^+^ versus PD-1^−^ B16-F10 WT melanoma cells or (**d**) respective activated WT (C57BL/6) T-cell subsets. All results are representative of at least *n* = 3 independent experiments. **p < 0.01; ND, not detected.

PD-1 surface protein expression on viable B16-F10 melanoma cells has been reported by several groups^5,7,12,16^. Nevertheless, one published report challenged PD-1 expression by melanoma cells by suggesting that the anti-PD-1 29F.1A12 ab clone, also used in previous studies^5,7^, reacts with dying, but not live B16-F10 cells^34^. Using this PD-1 ab clone and FVD employed in the aforementioned report to distinguish live versus dead cells, we confirmed surface PD-1 protein expression on both live (FVD^−^) and dead (FVD^+^) WT B16-F10 tumor cells (3.2 ± 10.8% vs. 6.6 ± 1.0%, mean ± s.e.m.,* n* = 10, respectively, Fig. 2a). As expected, 29F.1A12 showed markedly enhanced reactivity to respective PD-1 OE (82.3 ± 3.2% vs. 14.3 ± 4.0%, *n* = 4, Fig. 2a) and only minimal binding to live PD-1 KO B16-F10 melanoma variant cells (0.2 ± 0.0% vs. 0.5 ± 0.1%, *n* = 3, Fig. 2a). We also detected PD-1 surface protein expression on live positive control unactivated (6.0 ± 1.5%, *n* = 3) and activated WT T-cells (52.2 ± 7.9%, *n* = 6), but not at appreciable levels on live negative control PD-1 KO unactivated (0.0 ± 0.0%, *n* = 3) or activated T-cells (0.5 ± 0.1%, *n* = 3, Fig. 2b). 29F.1A12 showed increased reactivity with FVD^+^ WT and PD-1 KO T-cells (Fig. 2b), confirming PD-1 specificity of this anti-PD-1 ab clone for live, and lesser specificity for dead cells. To independently validate PD-1 recognition by 29F.1A12 on live cells, we FACS-purified PD-1^+^ versus PD-1^−^ subpopulations of FVD^−^ B16-F10 WT cells and activated WT T-cells and assessed *Pdcd1* gene expression as above. Compared to PD-1^−^ B16-F10 cells or activated WT T-cells, *Pdcd1* levels were > 19- or 6-fold enriched (*p* < 0.01) among PD-1^+^ melanoma cell fractions (Fig. 2c) or T-cell isolates (Fig. 2d), respectively. These findings show that the anti-PD-1 29F.1A12 ab clone recognizes surface PD-1 protein on live B16-F10 melanoma and WT T-cells.

### The anti-PD-1 RMP1-30 monoclonal antibody recognizes PD-1 surface protein on live B16-F10 melanoma cells

**Figure 3:**
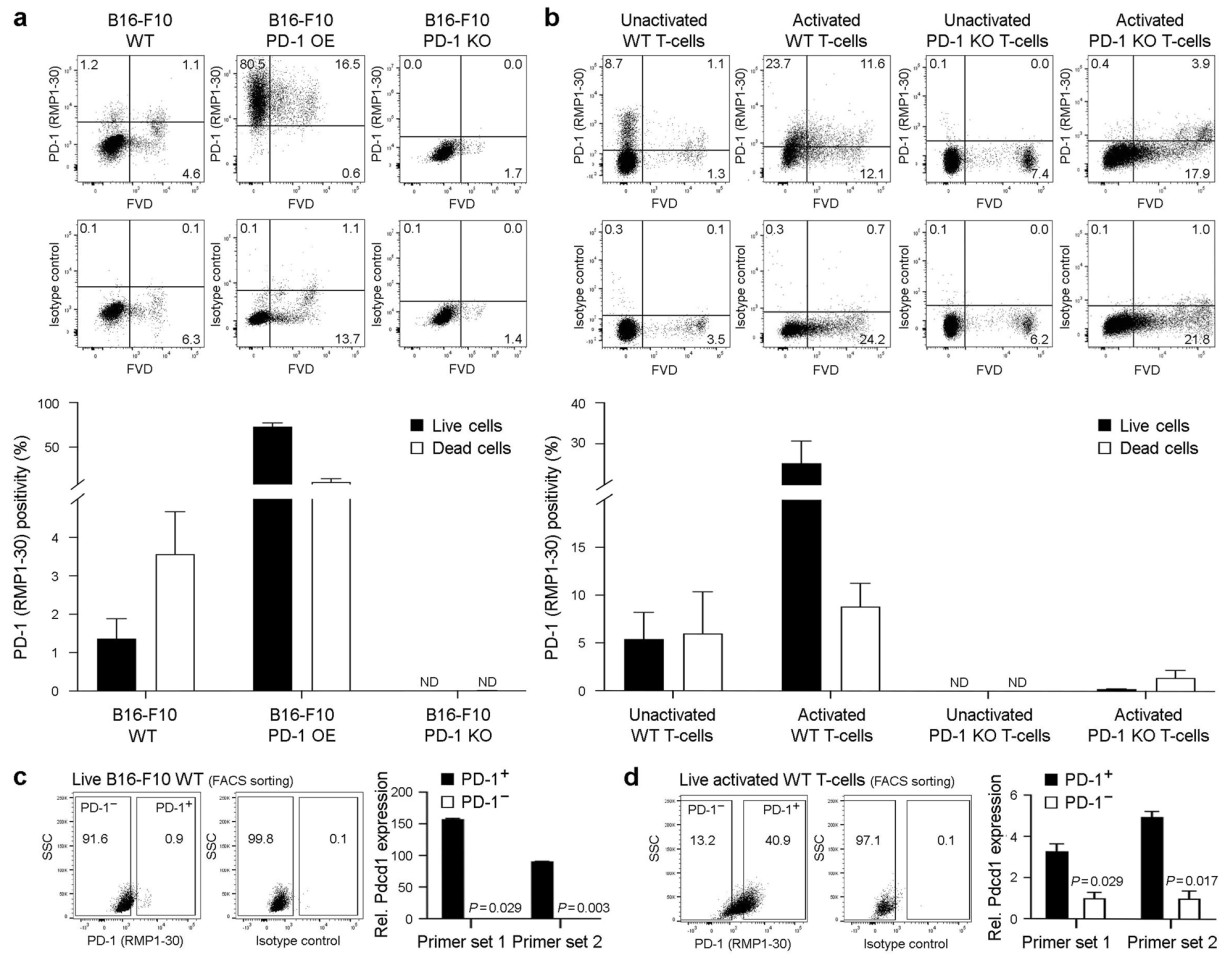
The anti-PD-1 RMP1-30 monoclonal antibody recognizes PD-1 surface protein on live B16-F10 melanoma cells. (**a,b**) Representative FACS plots of surface PD-1 protein expression (clone RMP1-30) or isotype control staining and % positivity (mean ± s.e.m.) on live versus dead (fixable viability dye, FVD-negative or -positive, respectively) (**a**) B16-F10 wild-type (WT), PD-1-overexpressing (OE), or PD-1 knockout (KO) melanoma cells, or on (**b**) unactivated versus activated WT or PD-1 KO T-cells (C57BL/6), respectively. (**c,d**) Relative Pdcd1 expression (mean ± s.d., two distinct primer sets), as determined by real-time qPCR (right), in FACS-sorted (RMP1-30, left) live (FVD^−^) (**c**) PD-1^+^ versus PD-1^−^ B16-F10 WT melanoma cells or (**d**) respective activated WT (C57BL/6) T-cell subsets. All results are representative of at least *n* = 3 independent experiments. *p < 0.01; **p < 0.01; ND, not detected.

To corroborate our findings of PD-1 surface protein expression on live B16-F10 melanoma cells using 29F.1A12, we employed an independent anti-PD-1 ab clone^4,37,38^, RMP1-30, as above. Flow cytometric analyses revealed RMP1-30 reactivity with both live (1.4 ± 0.5%, mean ± s.e.m., *n* = 6) and dead (3.6 ± 1.1%) WT B16-F10 melanoma cells, markedly enhanced binding to PD-1 OE (73.0 ± 4.1% vs. 10.7 ± 3.9%, *n* = 4, respectively, Fig. 3a), and no reactivity with PD-1 KO B16-F10 variants (0.0 ± 0.0% vs. 0.0 ± 0.0%, *n* = 3, respectively, Fig. 3a). As expected, RMP1-30 also recognized PD-1 surface protein on live unactivated (5.4 ± 2.8%, *n* = 3) and activated WT (25.4 ± 5.4%, *n* = 6), but not negative control PD-1 KO unactivated (0.0 ± 0.0%, *n* = 3) or activated T-cells (0.2 ± 0.0%, *n* = 3, Fig. 3b). RMP1-30 showed some binding to FVD^+^ WT and only minimal or no reactivity with respective PD-1 KO T-cells (Fig. 3b), thus demonstrating PD-1 specificity of this ab clone for both live and dead cells. *Pdcd1* transcript expression was markedly enriched (*p* < 0.05) among RMP1-30 FACS-sorted, PD-1^+^ versus PD-1^−^ B16-F10 WT cells (Fig. 3c) or activated WT T-cell cohorts (Fig. 3d). Together, these results confirm surface PD-1 protein expression on live B16-F10 WT melanoma cells, using two distinct anti-PD-1 ab clones, RMP1-30 and 29F.1A12.

### The 29F.1A12 and RMP1-30 anti-PD-1 antibody clones detect PD-1 surface protein on live YUMM melanoma variant lines

**Figure 4:**
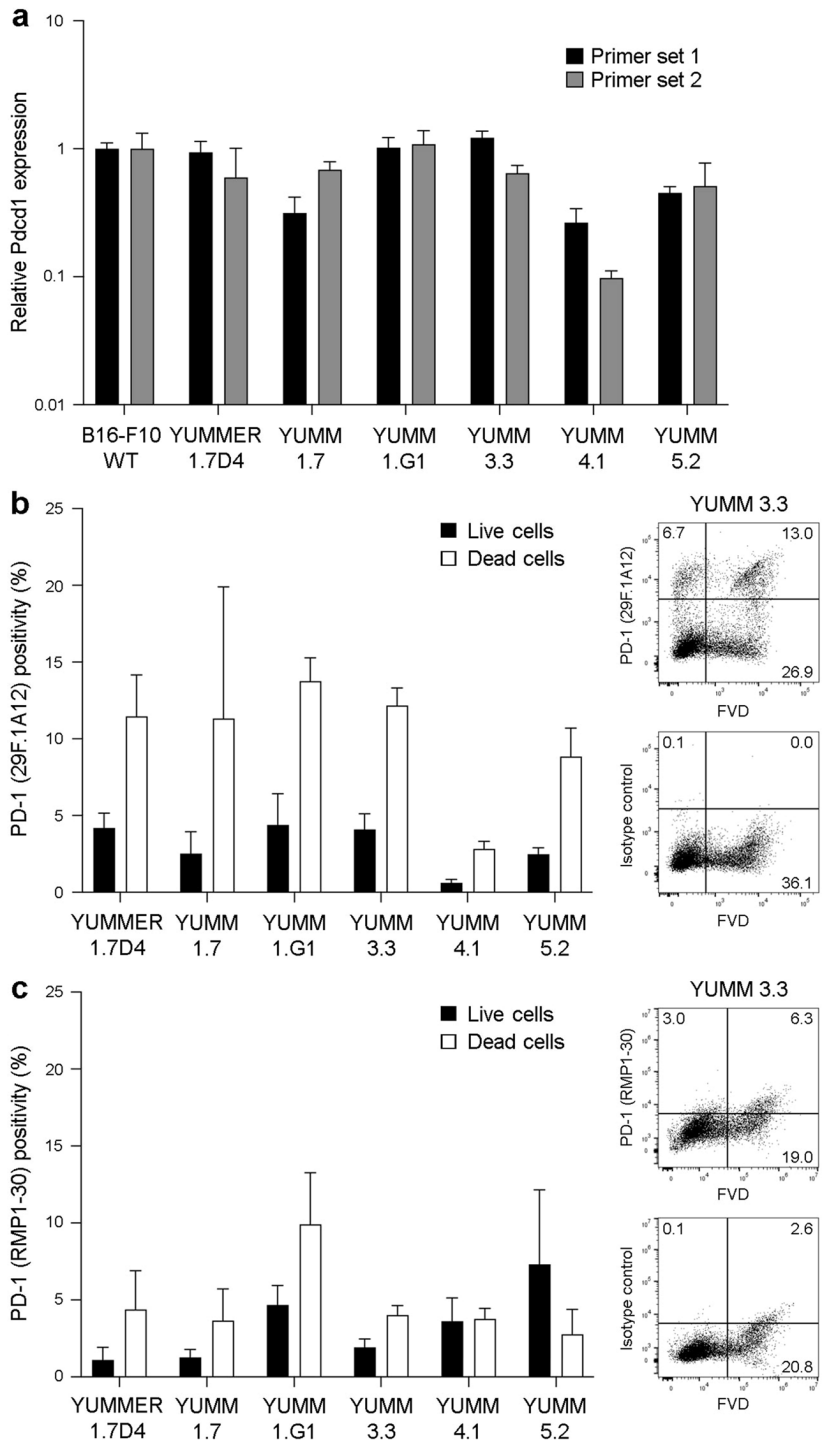
The anti-PD-1 29F.1A12 and RMP1-30 antibody clones recognize PD-1 surface protein on live YUMM melanoma cells. (**a**) Relative PD-1 gene (*Pdcd1*) expression (mean ± s.d., two distinct primer sets) in wild-type B16-F10, YUMMER1.7D4, YUMM1.7, YUMM1.G1, YUMM3.3, YUMM4.1, and YUMM5.2 cells. (**b,c**) Representative FACS plots (YUMM3.3) of surface PD-1 protein expression (clones 29F.1A12 and RMP1-30, respectively) or isotype control staining and % positivity (mean ± s.e.m.) on live versus dead (fixable viability dye, FVD-negative or -positive, respectively) YUMM melanoma cell lines, as in (**a**).

To evaluate the utility of both *Pdcd1* primer sets and anti-PD-1 FACS ab clones to detect tumor cell-intrinsic PD-1 across additional melanoma lines, we assessed PD-1 gene and protein expression in a series of YUMM murine melanoma variants with defined oncogenic mutations^35,36^. We detected *Pdcd1* expression in all six YUMM lines tested, YUMMER1.7D4, YUMM1.7, YUMM1.G1, YUMM3.3, YUMM4.1, and YUMM5.2, at levels that did not significantly differ from B16-F10 in 4 of 6 YUMM variants (Fig. 4a). Consistently, flow cytometric analysis revealed binding of 29F.1A12 (Fig. 4b) and RMP1-30 (Fig. 4c) to live, FVD^−^ YUMMER1.7D4 (4.2 ± 1.7% and 1.1 ± 1.4%, *n* = 3, respectively), YUMM1.7 (2.5 ± 2.5% and 1.3 ± 1.0%, *n* = 3), YUMM1.G1 (4.4 ± 3.5% and 4.7 ± 2.2%, *n* = 3), YUMM3.3 (4.1 ± 2.5% and 1.9 ± 1.1%, *n* = 4–6), YUMM4.1 (0.6 ± 0.4% and 3.6 ± 2.6%, *n* = 3), and YUMM5.2 cells (2.5 ± 0.9% and 7.3 ± 9.7%, *n* = 4–5). Similar to our findings in WT B16-F10 cells (Figs. 2, 3), 29F.1A12 and RMP1-30 also bound FVD^+^ YUMM cells (Fig. 4b,c). These results confirm PD-1 gene and protein expression by YUMM melanoma lines and further validate reagent utility for detecting tumor cell-intrinsic PD-1.

### The 29F.1A12 and RMP1-30 anti-PD-1 antibody clones recognize overlapping B16-F10 melanoma subpopulations

**Figure 5:**
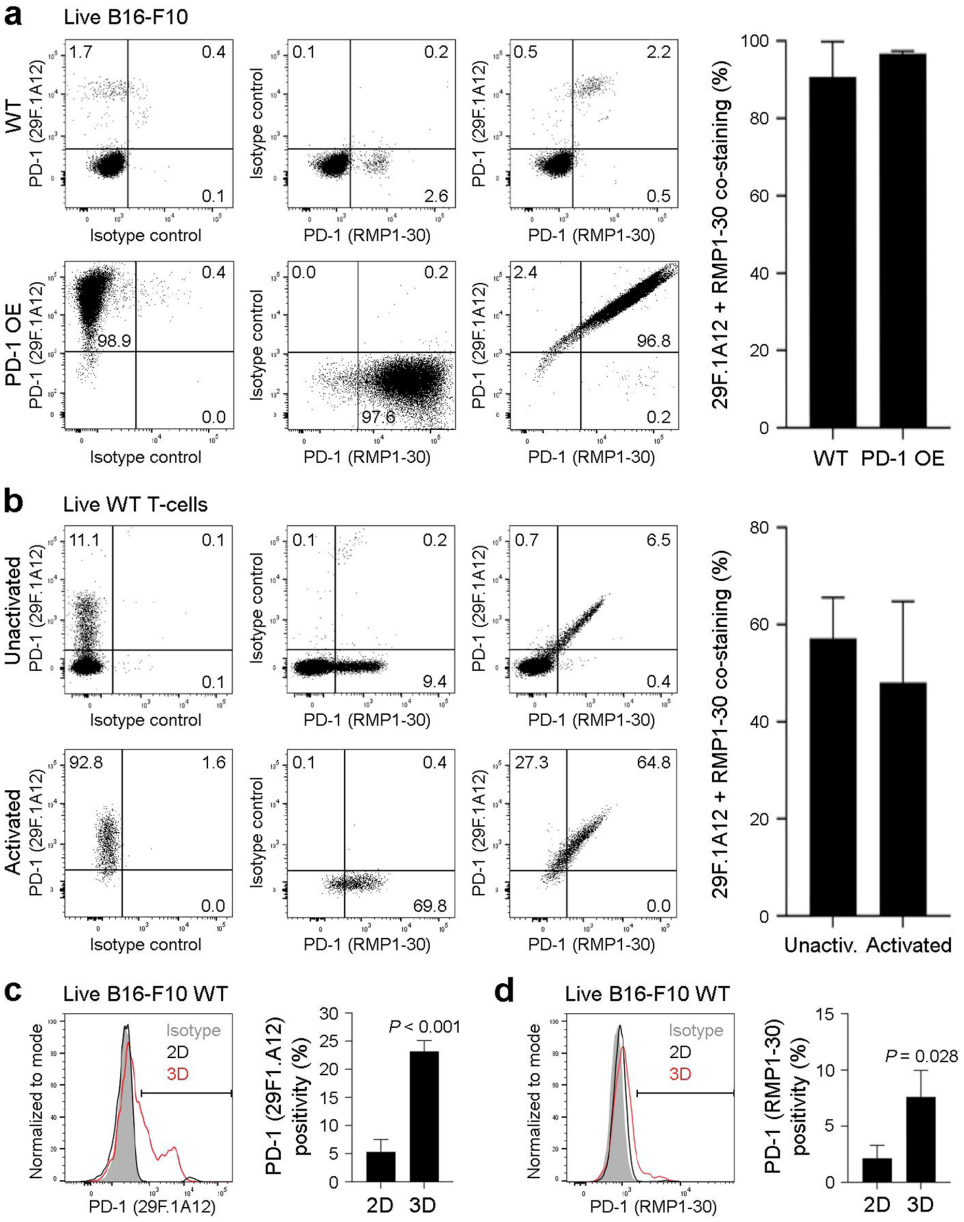
The 29F.1A12 and RMP1-30 anti-PD-1 antibody clones recognize overlapping B16-F10 melanoma subpopulations. (**a,b**) Representative FACS plots of 29F.1A12 and RMP1-30 co-staining (left) and % dual positivity (mean ± s.e,m., calculated as a fraction of RMP1-30-reactive cells) of PD-1 surface protein expression (right) by live (FVD^−^) (**a**) B16-F10 wild-type (WT) versus PD-1-overexpressing (OE) melanoma cells or (**b**) unactivated versus activated WT T-cells (C57BL/6) reveals co-localization of PD-1 antibody binding. (**c,d**) Representative histograms (left) and % positivity (mean ± s.e.m.) of PD-1 surface protein expression (right) by live (FVD^−^) B16-F10 WT cells grown in standard (2D, black line) versus tumor spheroid (3D, red line) culture conditions, as determined by the (**c**) 29F.1A12 or (**d**) RMP1-30 anti-mouse PD-1 antibody clones. Results are representative of at least *n* = 3 independent experiments. *p < 0.01; ***p < 0.001.

We next assessed whether 29F.1A12 and RMP1-30 recognize overlapping melanoma or T-cell subpopulations, to further confirm anti-PD-1 ab specificity. Co-staining with both ab clones revealed dual 29F.1A12/RMP1-30 positivity by nearly all PD-1 ab-reactive WT (90.8 ± 9.2%, mean ± s.e.m., *n* = 3) and PD-1 OE B16-F10 live (FVD^−^) melanoma cells (96.7 ± 0.7%, *n* = 3), as determined by flow cytometric analyses (Fig. 5a). Similarly, 29F.1A12 and RMP1-30 abs dually bound overlapping subpopulations of live YUMM cell line variants (not shown), unactivated (57.2 ± 8.4%) and activated WT T-cells (48.1 ± 16.7%, *n* = 4, Fig. 5b). Because the 29F.1A12 PD-1 blocking ab^4,8^ inhibits B16-F10 melanoma growth in three-dimensional (3D) tumor spheroid, but not standard two-dimensional (2D) cultures^5^, we next assessed if anti-PD-1 ab binding to WT B16-F10 cells was enhanced in 3D versus 2D conditions. Indeed, both 29F.1A12 and RMP1-30 reactivity to live WT B16-F10 cells was > 3-fold increased in 3D versus 2D cultures (Fig. 5c,d), consistent with variations in tumor cell-intrinsic PD-1 levels under distinct culture conditions reported by others^15,17,29^.

### The 29F.1A12 PD-1 blocking antibody inhibits PD-1 interactions with recombinant PD-L1 on live B16-F10 melanoma cells

**Figure 6:**
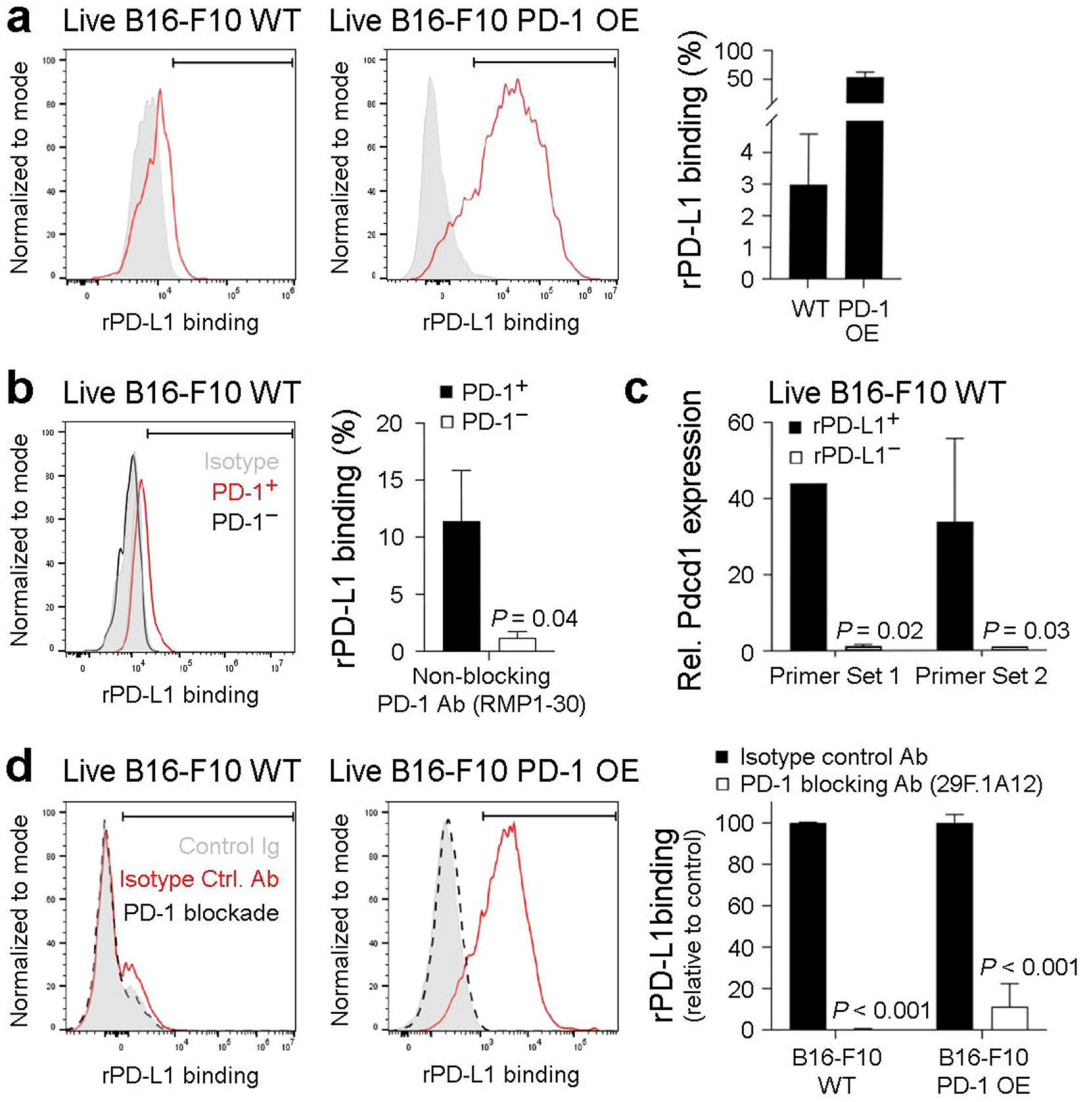
The 29F.1A12 anti-PD-1 monoclonal antibody blocks PD-1 interactions with recombinant PD-L1 on live B16-F10 melanoma cells. (**a,b**) Representative histograms (left) and % recombinant (r)PD-L1 binding (mean ± s.e.m., right), to live (FVD^−^) (**a**) B16-F10 wild-type (WT) or PD-1-overexpressing (OE) melanoma cells or (**b**) PD-1^+^ (red line) versus PD-1^−^ (black line), RMP1-30-gated B16-F10 WT melanoma cell subset. (**c**) Relative Pdcd1 expression (mean ± s.d., two distinct primer sets), as determined by real-time qPCR, in live (FVD^−^) FACS-sorted, rPD-L1^+/−^ B16-F10 WT subpopulations. (**d**) Effect of antibody-mediated PD-1 blockade (29F.1A12, black dashed line) versus isotype control (red line) on rPD-L1 binding to B16-F10 WT or PD-1 OE melanoma cells. Shown are representative histogram plots (left) and % rPD-L1 relative to isotype control (mean ± s.e.m., right). Results are representative of at least *n* = 3 independent experiments. *p < 0.01; ***p < 0.001.

To further confirm PD-1 functional expression on tumor cells, we assessed binding of recombinant (r) mouse PD-L1 to live WT and PD-1 OE B16-F10 melanoma cells. Flow cytometric analysis revealed binding of rPD-L1 to 3.0 ± 1.6% (mean ± s.e.m., *n* = 4) of live (FVD^−^) WT B16-F10 and > 50% reactivity with PD-1 OE cells (*n* = 4, Fig. 6a), consistent with PD-1 expression levels on respective cell variants (Figs. 2a, 3a). Co-staining with the non-blocking^37^ anti-PD-1 ab, RMP1-30, confirmed significantly greater rPD-L1 reactivity with PD-1^+^ (11.4 ± 4.5%, *n* = 3) compared to PD-1^−^ (1.2 ± 0.6%, *n* = 3) live WT B16-F10 tumor cell fractions (Fig. 6b). Consistently, *Pdcd1* expression was > 30-fold increased in rPD-L1-bound versus -nonreactive FACS-purified live WT B16-F10 subpopulations (Fig. 6c). Pre-treatment with the 29F.1A12 PD-1 blocking ab^4,8^ fully inhibited rPD-L1 binding to live WT B16-F10 cells (*n* = 3, *p* < 0.001) and neutralized rPD-L1 reactivity with PD-1 OE cells by > 90% (*n* = 3, *p* < 0.001, Fig. 6d). Together, these results confirm PD-1 functional expression by live B16-F10 melanoma cells using an ab-independent, rPD-L1-based approach and, for the first time, demonstrate tumor cell-PD-1:PD-L1 interactions.

## Discussion

In this study, we leveraged several independent methodologies, including qRT-PCR, immunoblotting, IP, and FACS using distinct primer sets, ab clones, rPD-L1, WT and PD-1 transgenic negative and positive control cell variants, to validate PD-1 functional expression by live B16-F10 and YUMM murine melanoma cells. Our results provide unequivocal evidence, including via MS-based sequencing, that PD-1 is expressed by B16-F10 cells. Moreover, we show, for the first time, binding of tumor cell-PD-1 to PD-L1 and the blockade of this interaction by the PD-1 neutralizing ab^4,8^, 29F.1A12. Consistently, several studies have reported PD-1 expression by B16-F10 cells^5,7,12,15,16^, including some that have used 29F1.A12^5,7^. Tumor cell-intrinsic PD-1 has also been found in murine non-small cell lung^20^, hepatocellular^24^, glioblastoma^23^, and ovarian cancer lines^7,12^ and in human melanoma^5,13–15,17–19^, lung^17,20,21^, liver^18,24–26^, brain^22,23^, colorectal^18,19,21^, pancreatic^17,27^, gastric^28^, esophageal^29–31^, and thyroid tumors^32,33^.

PD-1 reactivity varies by ab clone, assay system, cell type, and culture condition^15,17,29^. For example, while both 29F.1A12 and RMP1-30 clones detect surface PD-1 on overlapping WT murine B16-F10 and YUMM melanoma and T-cell subsets by FACS in this study, 29F.1A12 blocks rPD-L1 interactions in contrast to RMP1-30. Meanwhile, the RMP1-14 clone, but not 29F.1A12 or RMP1-30, is useful for pulling down (IP) and sequencing PD-1 from B16-F10 and T-cell cultures. It is well established that individual abs differ in their suitability for specific assay types. Posttranslational modifications (PTM) of the PD-1 protein can alter PD-1 expression and accessibility of ab binding epitopes^18,39–41^. Indeed, PD-1 bears multiple N-linked glycans^42,43^, some of which are critical for ab and PD-L1 binding, as defined in human T-cells^40,41,44,45^. Because glycosylation often differs between cell types, species, and even cell lines of similar etiology^46^, glycostructures should be considered as variables in PD-1 detection efficiency by individual ab clones, including in B16-F10 and YUMM cells.

Culture conditions can greatly affect PD-1 ab clone reactivity. For example, we found that 29F.1A12 and RMP1-30 binding to B16-F10 cells was increased in 3D versus 2D settings. This result is consistent with our prior demonstration of B16-F10 growth inhibition in 3D tumor spheroid, but not standard 2D, cultures^5^, and with work by others revealing PD-1 induction in B16-F10 cells exposed to hypoxia^15^, a condition prevalent within tumor spheroids^47^. Defining additional factors regulating cell type-specific PD-1 expression level, PTM, and ab recognition will require a future dedicated effort. Nevertheless, our study contributes to the growing body of evidence that tumor cell-PD-1 detection can substantially increase depending on environmental cues^13,15,17,18,29^.

Although multiple groups have identified PD-1 on diverse cancer cell types, including B16-F10^5,7,12,15,16^, one study surprisingly claims that B16-F10 cells are negative for PD-1, and that PD-1 ab clones, such as 29F.1A12, exclusively bind to an off-target nuclear antigen exposed by dead cells^34^. Unfortunately, this work has shortcomings in experimental design and data interpretation, incompletely describes methodological details, and fails to reference already published studies demonstrating PD-1 expression by live B16-F10 cells^7,12^. First, Metzger et al.^34^ employed PD-1 abs at an inadequately low concentration of 1 µg/mL (1:200 dilution of the 200 µg/mL 29F.1A12 ab stock) for FACS-based PD-1 detection on B16-F10 cells. This ab amount is > 10-fold lower than previously reported concentrations (10–20 µg/mL) used for staining tumor cell-PD-1^5^ and also than clinical PD-1 ab serum titers (> 10–100 µg/mL) achieved in patients at time of administration^48–50^. Absent throughout the manuscript were matched isotype control ab FACS plots. We used PD-1 abs at 10–20 µg/mL, proper isotype-controlled gating strategies, and the identical viability dye^34^, and found significant binding of 29F.1A12 and RMP1-30 to overlapping live (FVD^−^) B16-F10 and YUMM subpopulations, in contrast to the study in question. We also observed PD-1 ab clone reactivity with dead (FVD^+^) melanoma cells, as reported^34^. Nonetheless, such ab binding cannot be exclusively ascribed to off-target recognition of a nuclear antigen in dead cells^34^ because both 29F.1A12 and RMP1-30 reacted more avidly with FVD^+^ WT versus PD-1 KO B16-F10 melanoma or T-cells, which differ in PD-1 but not nuclear antigen content. Our findings thus indicate that both ab clones detect PD-1, including in dead cells. On target PD-1 ab binding was further supported by the fact that non-neutralizing^37^ RMP1-30 reactivity coincided with, while 29F.1A12 fully blocked, rPD-L1 ligation to B16-F10 cells.

Second, Metzger et al.^34^ do not describe *Pdcd1* primer sequences and qRT-PCR amplification conditions for detecting PD-1 gene expression in B16-F10 cells. Moreover, the authors omit crucial PD-1 KO negative controls in gene expression analyses that were otherwise prevalently employed elsewhere. The apparent absence of an electrophoresis band for B16-F10 cells^34^ does not exclude *Pdcd1* amplification by qRT-PCR. Indeed, a short 77 bp fragment^34^ is significantly harder to detect than products of greater length, due to decreased dye incorporation and corresponding emission. This is particularly true when product levels are low and agarose gels are used at an atypically high concentration (3%)^34^ resulting in increased opacity. Our qRT-PCR analyses rigorously confirmed PD-1 gene expression by WT B16-F10 cells using two independent *Pdcd1* primer sets with specified sequences and amplification settings, and multiple control cells of defined PD-1 expression level, including PD-1 KO, OE, WT, and PD-1^+^ versus PD-1^−^ FACS (29F.1A12, RMP1-30, rPD-L1)-purified B16-F10 and T-cell cohorts. It should be noted that nucleotide or amino acid sequencing methodologies represent gold standards for validating expression of a gene or protein of interest, respectively. Indeed, we previously amplified and sequenced the full *Pdcd1* coding region from B16-F10 cells (GenBank accession KJ865858)^5^. In the current study, we confirmed expression of PD-1 protein in B16-F10 lysates by IP and MS-based sequencing. Together, our work and that of others^7,12,15,16^ therefore unequivocally establishes PD-1 expression by B16-F10 cells.

The scientific method encourages spirited debate based on empirical evidence. Replication of results is a pivotal hallmark of knowledge advancement. Reproducibility of research data requires well-defined experimental conditions, careful controls, and an open dialogue between groups to reconcile apparent discrepancies in results. In these respects, the manuscript by Metzger and colleagues^34^ rather fell short, as elaborated above. The inability to detect PD-1, or any molecule for that matter, does not definitively exclude its expression, particularly when using conditions distinct from other reports. Moreover, findings restricted to a single cell line, B16-F10, cannot be extrapolated to rule out PD-1 expression by all other tumor cells and even non-T immune cell lineages, as overstated by the authors^34^. Such unsubstantiated conclusions can hinder the advancement of science, especially when they dismiss the contributions of countless groups.

We appreciate the challenges inherent to studying tumor cell-intrinsic PD-1, because its expression is often restricted to subsets of cancer cells^5,7,12,14,19,20,23^ and may vary by ab clone, assay type, and culture condition^13,15,17,18,29^. Nevertheless, there is growing appreciation of tumor cell-intrinsic PD-1 roles in tumorigenesis and response to immune checkpoint therapy across multiple malignancies^5,7,15,17,19–21,23–25,27,28,32^. Accordingly, validating reagents, defining assay systems and the experimental conditions that enable mechanistic dissection of cancer cell-PD-1 checkpoint immunobiology are important endeavors. This study represents a crucial step forward in this regard.

## Methods

### Cell culture

Murine B16-F10, YUMM1.7, YUMM1.G1, YUMM3.3, YUMM4.1, and YUMM5.2 melanoma cells were newly purchased from ATCC (Gaithersburg, MD), and YUMMER1.7D4 from Sigma (St. Louis, MO). All melanoma lines used were at low passage, < 70% confluency, and maintained in RPMI-1640 medium (Life Technologies, Carlsbad, CA) supplemented with 10% (v/v) fetal bovine serum (FBS, Sigma) and 1% (v/v) penicillin/streptomycin (Life Technologies) in standard culture flasks for 2D expansion or in 6-well ultra-low attachment plates (Corning, Glendale, AZ) for 3D tumor spheroid culture, as described^5^. PD-1 (*Pdcd1*) OE and vector control B16-F10 melanoma cells were generated previously^5^ and cultured in the presence of 1 μg/mL puromycin (Life Technologies) and 500 μg/mL neomycin (G418 sulfate, Life Technologies). PD-1^−/−^ knockout (KO) B16-F10 melanoma cells were generated and validated as described below. Cells grown in 2D were harvested using 0.1% (v/v) versene solution (Life Technologies), as described^5^, and 3D tumor spheroids were dissociated into single cell suspension after 5 days in culture for subsequent flow cytometric analysis using enzyme-free dissociation buffer (Thermo Fisher, Waltham, MA), per the manufacturer’s instructions.

### Mice

C57BL/6 mice were purchased from The Jackson Laboratory (Bar Harbor, ME) and PD-1^−/−^ KO C57BL/6 mice^51^ maintained and housed at the Brigham and Women’s Hospital (BWH) animal facility, as described^5,52^. All mice were female, at least 6 weeks of age, and used in accordance with the National Institutes of Animal Healthcare Guidelines under protocol 2016N000112 approved by the Institutional Animal Care and Use Committee of BWH. The study is reported in accordance with ARRIVE guidelines.

### Isolation and activation of mouse T-cells

Splenocytes were isolated from mouse spleens, as described^51,52^. Specifically, single cell suspensions were obtained by mechanical disruption of spleens and filtered through a 70 µm cell strainer (BD Biosciences, Franklin Lakes, NJ). Red blood cells were removed using the hypotonic ammonium chloride ACK lysing buffer (Life Technologies) per the manufacturer’s instructions. Splenocytes were then resuspended at 1 × 10^6^ cells/mL in advanced RPMI 1640 medium supplemented with 1% penicillin/streptomycin, 1% GlutaMAX, 10 mM HEPES (all from Life Technologies), 10% heat-inactivated FBS (Sigma), 30 IU/mL of rmIL-2 (BioLegend, San Diego, CA), and 2 μg/mL of soluble anti-mouse CD28 ab (37.51, BD Biosciences, Woburn, MA), and seeded in 96-well plates (Corning) pre-coated with 10 μg/mL of anti-mouse CD3 ab (145-2C11, BD Biosciences)^52^. Cells were activated for 5 to 6 days prior to subsequent study.

### RNA extraction and real-time quantitative RT-PCR analysis

Total RNA was isolated using the RNeasy Micro Kit or the RNeasy Plus Mini Kit (Qiagen, Germantown, MD), according to the manufacturer’s protocol. RNA was subsequently converted to cDNA using the SuperScript VILO cDNA synthesis kit (Thermo Fisher), and samples assayed in triplicate using the Fast SYBR Green Master Mix (ThermoFisher) with primer sets, as below, on a QuantStudio 5 Real-Time PCR system (Applied Biosystems, Waltham, MA). Thermal cycling was carried out at 94 °C for 2 min, followed by 40 cycles at 94 °C for 15 s, 60 °C for 20 s and 68 °C for 1 min, as described^5^, followed by melt curve validation of amplicons. Data was normalized to murine actin, and relative transcript levels calculated using the delta-delta Ct method. Samples with threshold cycle (Ct) numbers above the water negative control were designated as not detected. The primer sequences used for murine *Pdcd1* detection were: primer set 1, forward-5′-CGGTTTCAAGGCATGGTCATTGG-3′, reverse-5′- TCAGAGTGTCGTCCTTGCT TCC-3′; primer set 2, forward-5′-GGAGCAGAGCTCGTGGTAAC-3′, reverse-5′-AATGACCATGCCTTGAAACC-3′. For murine actin, primer sequences were: forward-5′-CATCGTACTCCTGCTTGCTG-3′ and reverse-5′-AGCGCAAGTACT CTGTGTGG -3′.

### Generation of PD-1 KO B16-F10 melanoma cells

B16-F10 PD-1 KO cells were created using CRISPR/Cas-9 by inserting the guide (g) RNA, GAGCAGAGCTCGTGGTAAC (ThermoFisher), targeting murine *Pdcd1* into the vector pKLV2-U6gRNA5(BbsI)-PGKpuro2ABFP-W (67,974, Addgene, Watertown, MA). We first generated a monogenetic founder B16-F10 tumor cell clone by single cell sorting. The B16-F10 founder cells were co-transfected with the above construct and with Cas9-EGFP (LentiCas9-EGFP, plasmid #63,592, Addgene). Cells were then doubly selected 1–2 days post-transfection in media containing puromycin (5 μg/ml, Life Technologies) and blasticidin (20 μg/ml, ThermoFisher) over 3–5 days, and sorted for BFP and EGFP expression. The B16-F10 transfectant pool was expanded for 3–5 days and then subcultured into 96-well plates by limiting dilution to generate single cell clones. Colonies were determined by visual inspection, expanded and screened for loss of PD-1 gene and protein expression by real-time quantitative RT-PCR, flow cytometry, and immunoblotting, as described herein. To confirm chromosomal knockout of PD-1, genomic DNA was isolated from candidate clones (DNeasy Blood & Tissue Kit, Qiagen), and the *Pdcd1* locus was PCR-amplified (Platinum Taq DNA Polymerase, ThermoFisher) with primers bracketing the *Pdcd1* gRNA binding site. Thermal cycling was carried out in the presence of 1.5 mM MgCl_2_ at 95 °C for 30 s, followed by 30 cycles at 95 °C for 20 s, 63 °C for 20 s, and 68 °C for 30 s, with a 10 min final extension at 68 °C. The primer sequences used were forward-5′-GATGCCCGCTTCCAGATCATA-3′ and reverse-5′-AGAGCCTAAGAGGTCTCTGGG -3′. The 222 bp PCR products reflecting individual alleles were gel-purified, ligated into pCR2.1 (TA Cloning Kit, ThermoFisher), and transformed into One Shot TOP10 Chemically Competent E. coli (ThermoFisher). Plasmids were purified (ZymoPURE Plasmid Miniprep Kit, Zymo Research, Irvine, CA), sequenced (Azenta Life Sciences, Chelmsford, MA), and homozygous disruption of *Pdcd1* confirmed.

### Antibodies and reagents

The following abs and reagents were used for flow cytometric analysis: Fixable Viability Dye eFluor 780 (FVDeF780, Invitrogen, Waltham, MA)^34^, TruStain FcX PLUS Fc Receptor Blocking Solution, fluorescein isothiocyanate (FITC)- or phycoerythrin (PE)-conjugated anti-mouse PD-1 ab (29F.1A12)^4,5,7,37^, PerCP Cy5.5- or allophycocyanine (APC)-conjugated anti-mouse PD-1 ab (RMP1-30)^4,37,38^, PE-Cy7-conjugated anti-mouse CD3 ab (17A2), FITC- or PE-conjugated rat IgG2a (RTK2758), and PerCP Cy5.5- or APC-conjugated rat IgG2b (RTK4530) isotype control abs (all from BioLegend), recombinant mouse PD-L1/B7-H1 Fc chimera protein (rPD-L1)^5^ and rhIgG1 Fc protein (R&D Systems, Minneapolis, MN), and FITC-conjugated anti-human IgG1 ab (Abcam, Cambridge, MA). For IP studies, we used: rat-anti-mouse PD-1 (RMP1–14)^53^ and rat IgG2a isotype control abs (Invitrogen). For immunoblotting the following reagents were used: goat anti-mouse PD-1 ab (AF1021, R&D Systems), anti-actin ab-5 (clone C4, BD Biosciences), HRP-conjugated goat anti-mouse IgG and donkey anti-goat IgG abs (Southern Biotech, Birmingham, AL).

### Flow cytometric analysis

Flow cytometric analyses of B16-F10, YUMM, and positive control T-cells (CD3-gated) were performed as described previously^5,14,52^. To exclude dead cells, FVDeF780 staining (1:1000) was performed in PBS for 30 min at 4 °C in the dark, following the manufacturer’s recommendations. Cells were then blocked with TruStain FcX PLUS for 10 min on ice, and then stained with fluorochrome-conjugated abs (10–20 μg/mL) described above, or with rPD-L1 (20–50 μg/mL) in PBS + 2% (v/v) FBS for 30 min at 4 °C. For assessment of RMP1-30 co-expression with rPD-L1 or of 29F.1A12-mediated blockade of rPD-L1 binding, B16-F10 cells were first incubated with anti-PD-1 ab for 30 min at 4 °C and then stained with rPD-L1, followed by counterstaining with FITC-anti-human IgG1 (10 µg/mL). Isotype control abs were employed and cell doublets excluded for all analyses. Fluorescence emission was acquired on a FacsCanto (BD Biosciences) or an Aurora Spectral Viewer (Cytek, Fremont, CA), and data analyzed using FlowJo version 10.8.1 (TreeStar, Ashland, OR).

### FACS sorting

B16-F10 cells or activated WT T-cells were harvested, washed, and incubated with FVDeF780, TruStain FcX PLUS, rPD-L1, anti-PD-1, anti-CD-3, or respective control abs, as above. 29F.1A12^+/−^, RMP1-30^+/−^, or rPD-L1^+/−^ cohorts negative for FVDeF780 were directly sorted into RLT Plus lysis buffer (Qiagen) supplemented with β-mercaptoethanol (Sigma) using a BD FACS Aria™ II cell sorter system, for subsequent qRT-PCR analyses as described above.

### Immunoblotting

Cells were lysed in ice-cold RIPA buffer supplemented with protease inhibitor cocktail (Roche, Basel, Switzerland) and vortexed at 4 °C for 30 min, as described^5^. Lysates were centrifuged and protein concentrations measured using the BCA protein assay kit (Thermo Fisher), following the manufacturer’s instructions. B16-F10 lysates were boiled in reducing Laemmli sample buffer (Bio-Rad, Hercules, CA) for 7 min, resolved in 7.5% SDS-PAGE gels (Bio-Rad) and proteins transferred to Sequi-Blot PVDF membranes (Bio-Rad). Membranes were blocked in tris-buffered saline (TBS, Boston BioProducts, Milford, MA) containing 5% (w/v) bovine serum albumin (BSA, Sigma) and 0.1% (v/v) Tween-20 (Sigma), for at least 1 h at room temperature (RT), and then incubated overnight at 4 °C with primary ab (10–20 μg/mL), followed by washing and incubation with secondary HRP-conjugated ab (1:1000) for 1 h at RT. Antigens were visualized using the Lumi-Light Western blotting substrate (Roche) on HyBlot CL Autoradiography Films (Thomas Scientific, Swedesboro, NJ) using a Kodak Min-R mammography processor (Kodak, Rochester, NY). For detection of actin, blots were stripped with Restore Western blot Stripping Buffer (Thermo Fisher) according to the manufacturer’s protocol, blocked, and incubated for 1–12 h at 4 °C with primary ab (1:1000–2500), and then with secondary HRP-goat anti-mouse ab (1:2500) for 1 h at RT.

### Immunoprecipitation

PD-1 IP studies were performed using Pierce Protein A/G Plus agarose beads (Invitrogen), according to the manufacturer’s instructions. Briefly, cells were lysed in ice-cold buffer (150 mM NaCl, 50 mM Tris-HCI and protease inhibitor cocktail, Roche), sonicated (three 10 s bursts), vortexed for 2 h at 4 °C in 2% Nonidet P-40 (NP-40, Sigma), and centrifuged. B16-F10 lysates were concentrated using Microcon-10 Ultracel PL-10 filter columns, as above. Cell lysates were precleared for 2 h at 4 °C by incubation with Protein A/G Plus agarose beads previously blocked in ice-cold buffer supplemented with 1% BSA for 1 h at 4 °C, incubated with anti-mouse PD-1 (4–6 µg, RMP1-14)^53^ or rat IgG2a isotype control abs for 2 h at 4 °C, and then with Protein A/G Plus agarose beads overnight at 4 °C under continuous rotation. Supernatants were kept for assessment of IP efficiency, Protein A/G Plus agarose beads washed extensively, and IP products eluted in ice-cold buffer, as above, supplemented with 1.5 × Non-reducing Lane Marker Sample Buffer (Thermo Fisher), and boiled at 100 °C for 7min^54^. IP products were then analyzed by immunoblotting, as above, or resolved in 7.5% SDS-PAGE gels, stained with Colloidal Coomassie Blue (Bio-Rad), and subjected to MS sequencing, as described below.

### Mass spectrometry

SDS/PAGE gels (7.5%) containing IP products were run at ~ 120 V. Gels were fixed in 40% (v/v) ethanol, 10% (v/v) acetic acid, 50% high-grade water for 15 min, and then stained with Colloidal Coomassie Blue overnight at RT, following the manufacturer’s instructions. Gel slices containing counterstained protein bands that coincided with the expected PD-1 molecular weight of ~ 32–55 kDa were excised, washed twice in 50% (v/v) acetonitrile, 50% (v/v) high-grade water. Gel slices were then submitted for peptide mass fingerprinting on a Q Exactive™ HF-X Hybrid Quadrupole-Orbitrap™ MS system (Thermo Fisher), as described^55^.

### Statistics

Experimental groups were compared statistically using the PRISM 9.0 software (GraphPad, San Diego, CA). The Student’s *t* test was used to compare two experimental groups, and one-way ANOVA with Dunnett post-test for comparison of three experimental groups, with *p* < 0.05 considered statistically significant. Data was tested for normal distribution using the D'Agostino and Pearson omnibus normality test.

See also the Supplementary Information.

## Supplementary Information

Supplementary Information.

## Data Availability

The data generated in this study is available from the corresponding authors upon reasonable request.
