# Supplementary material for: Distinct antibody clones detect PD-1 checkpoint expression and block PD-L1 interactions on live murine melanoma cells
Source: Sci Rep. 2022 Jul 21;12:12491. doi: 10.1038/s41598-022-16776-1 (PMC9304406; doi:10.1038/s41598-022-16776-1)

Supplementary Information

Full-length Western Blot Images for Figure 1b

B16-F10 melanoma cells – PD-1

WT PD-1 PD-1
OE KO

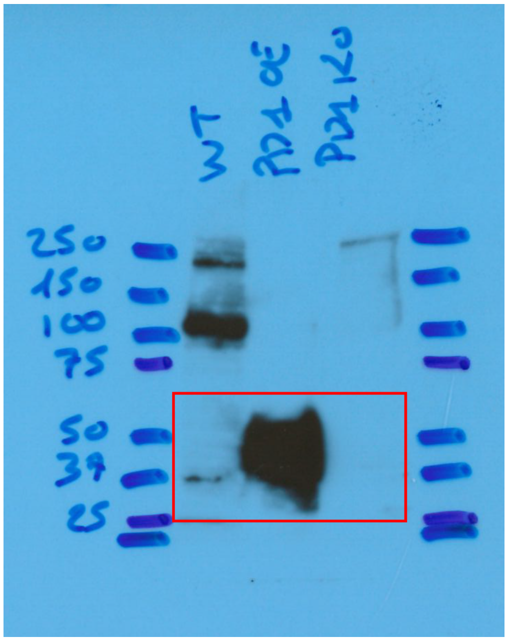

T-cells – PD-1

Unact. Act. Act.
WT WT PD-1 KO

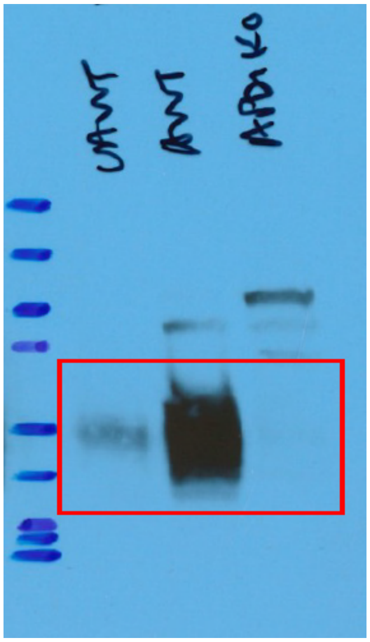

B16-F10 melanoma cells – Actin

WT PD-1 PD-1
OE KO

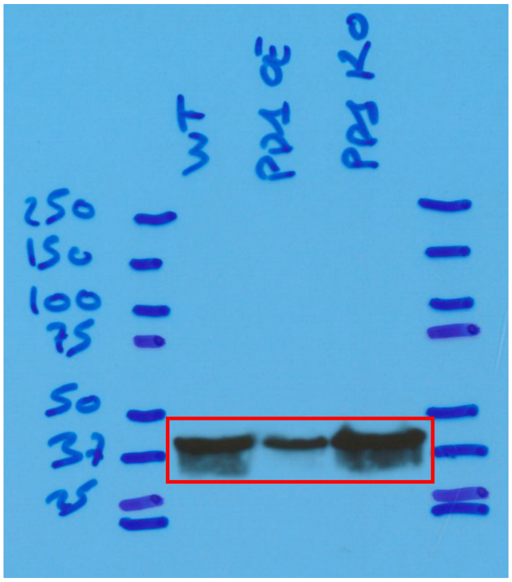

T-cells – Actin

Unact. Act. Act.
WT WT PD-1 KO

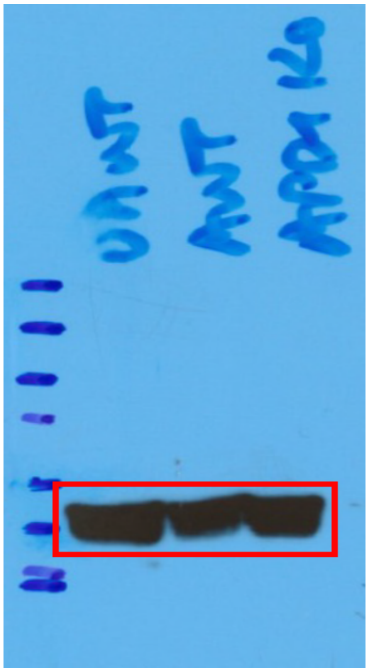

Full-length Western IP Images for Figure 1c

B16-F10 WT – PD-1

Total PD-1 IP SN PD-1 IP Iso IP

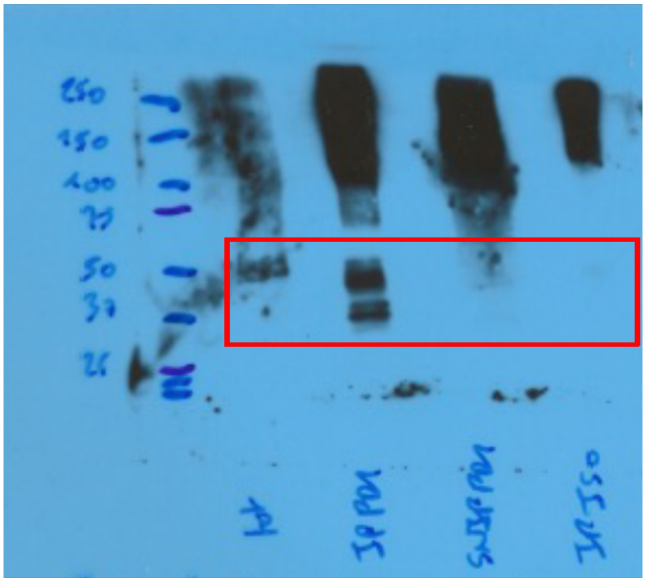

B16-F10 PD-1 OE – PD-1

Total PD-1 IP SN PD-1 IP Iso IP

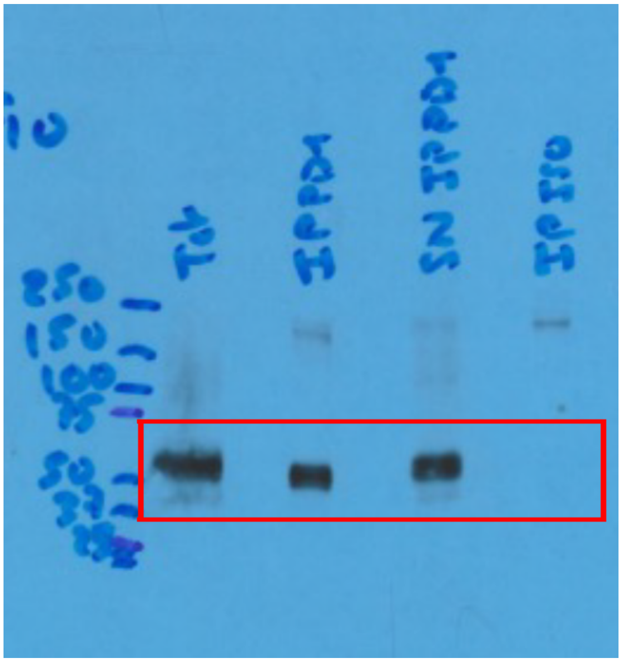

Activated WT T-cells – PD-1

Total PD-1 IP SN PD-1 IP Iso IP

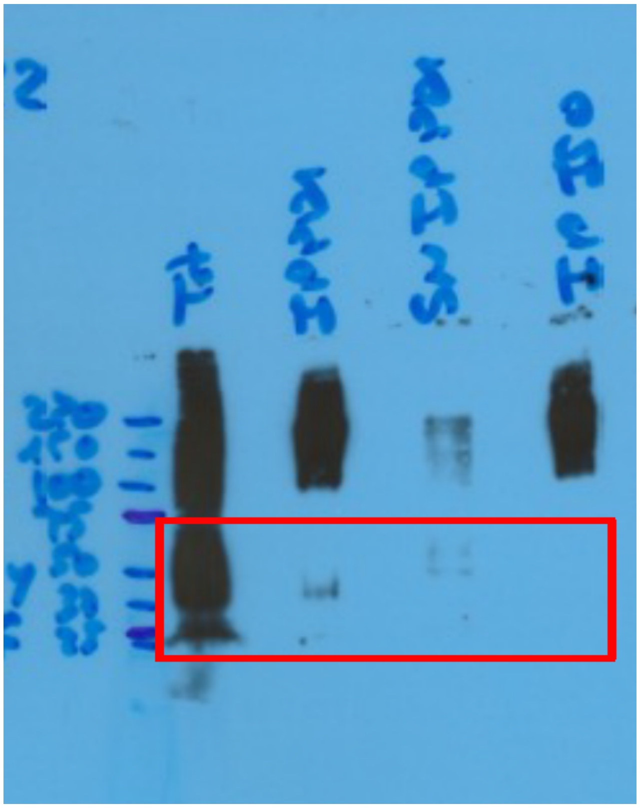

Supplement: Supplementary file 1 — Supplementary Information. [file 41598_2022_16776_MOESM1_ESM.pdf]
